# Prevalence of primary dysmenorrhea, its intensity, impact and associated factors among female students’ at Gondar town preparatory school, Northwest Ethiopia

**DOI:** 10.1186/s12905-019-0873-4

**Published:** 2020-01-06

**Authors:** Abere Woretaw Azagew, Destaye Guadie Kassie, Tarkie Abebe Walle

**Affiliations:** 1 Department of Medical Nursing, School of Nursing, College of Medicine and Health Sciences, University of Gondar, Gondar, Ethiopia; 2 Department of Pediatrics and Child Health Nursing, School of Nursing, College of Medicine and Health Sciences, University of Gondar, Gondar, Ethiopia; 3 Department of Surgical Nursing, School of Nursing, College of Medicine and Health Sciences, University of Gondar, Gondar, Ethiopia

**Keywords:** Female, Impact, Intensity, Primary dysmenorrhea, Prevalence, Preparatory school students

## Abstract

**Background:**

Primary dysmenorrhea is defined as a crampy pain in the lower abdomen before or during the menstrual period in the absence of any pelvic pathology. It is the leading motherhood problem worldwide but there is limited evidence on the prevalence of primary dysmenorrhea in the study area as well in Ethiopia. Researching primary dysmenorrhea helps to focus on the treatment plan. The study aimed to assess the prevalence, intensity, impact, and associated factors of primary dysmenorrhea among female students at Gondar town preparatory school.

**Methods:**

A cross-sectional study design conducted among female students at Gondar town Preparatory School from May 1–10/2017. A total of 459 study participants were used. A simple random sampling technique was used to select study participants. A self-administered structured questionnaire was employed. Epi Info version 7 and SPSS version 20 were used for data entry and analysis respectively. A binary logistic regression model was computed. Variables having a *p*-value < 0.05 in the multivariate logistic regression model were considered as statistically significant.

**Results:**

A total of 459 female students participated in the study with a response rate of 96.29%. The prevalence of primary dysmenorrhea among female students was found to be 64.7% (95% CI; 60.2–69.2%). Around 61% reported moderate intensity of menstrual pain and 50.7% complain about lower abdominal pain. Sixty-five percent of study participants reported that absenteeism from school was the impact of menstrual pain. Having irregular monthly menstrual cycle (AOR = 1.70, 95% CI; 1.02, 2.84) and positive family history of dysmenorrhea (AOR = 5.19, 95% CI: 3.21, 8.37) were significantly associated with primary dysmenorrhea.

**Conclusions:**

The prevalence of primary dysmenorrhea was found to be high. Having an irregular monthly menstrual cycle and a positive family history of dysmenorrhea were determinants of primary dysmenorrhea.

## Background

Dysmenorrhea is pain associated with menstruation. It is the most commonly reported menstrual disorder in women. More than half of women who menstruate have pain for 1–2 days each month [1, 2]. It is an extremely common and sometimes debilitating condition for women of reproductive age [3]. Its prevalence varies from between16 to 91% in reproductive age women [4]. Dysmenorrhea is classified as primary and secondary dysmenorrhea. Secondary dysmenorrhea is caused by a disease or condition such as infection, ovarian cyst, and endometriosis [5].

Primary dysmenorrhea is defined as cramping pain in the lower abdomen before or during the menstruation period in the absence of any pelvic pathology [6]. It is the leading women hood problem that affects 90% of adolescent girls and more than 50% menstruating women [7]. The pathophysiology of primary dysmenorrhea is due to increased and/or abnormal uterine activity as a result of increased production and release of prostaglandins [8, 9]. The clinical feature of Primary Dysmenorrhea is frequent and crampy pain which mainly affects the lower abdomen and radiates to the back or thigh [6].

Fatigue emotional disturbance, abdominal distension, nausea, vomiting, and sleep disturbances are associated with symptoms of dysmenorrhea [4]. The causes of primary dysmenorhea were not well studied, but the common risk factors are a positive family history of dysmenorrhea, not use of oral contraceptive, smoking, higher severity of bleedings, shorter/longer menstrual period interval, stress, and menstrual cycle irregularity [1, 2, 4, 10].

Daily activity limitation, absenteeism from school/work, social withdrawal, decrease academic performance, and increased health care medical costs are the negative effect of primary dysmenorrhea [4, 10–14]. Use of oral contraceptives [3], non-steroidal anti-inflammatory medications [15, 16], herbal medicines [17], massage [18], and lifestyle modification [19] are treatment strategies to reduce dysmenorrheic pain.

In Ethiopia, there is limited evidence on the prevalence, intensity, impact, and associated factors of primary dysmenorrhea among preparatory school female students in the study area.

## Methods

### Study design and settings

A cross-sectional survey was conducted among female preparatory school students in Gondar town from May 1–10/2017. Gondar town is one of the historical towns in Ethiopia. It is found in Amhara regional state, Northwest part of Ethiopia. It is about 750 km away from Addis Ababa the capital city of Ethiopia. In Gondar town, there are five preparatory schools of which three preparatory schools were selected.

### Source and study population

Female students who undergo their education in Gondar town preparatory school were considered as source population whereas female students who were present in the selected preparatory school during the data collection period were taken as the study population.

### Inclusion / exclusion criteria

Female students who undergo their education in the selected preparatory school at Gondar town were included in the study whereas female students who had a known diagnosed medical history of pelvic pathology were excluded in the study.

### Sample size and sampling procedures

The sample size was determined by using a single population proportion formula using the assumption of; 95% level of confidence, 4% marginal error and by taking the prevalence of dysmenorrhea from a previous study (77.6%) [12]. With these assumptions, the sample size became 417. Anticipating a10% nonresponsive rate, the required sample size was 459.The study participants were taken from the selected (Azezo, Fasiledes, and Angereb) preparatory schools using stratified random sampling with proportional allocation. The study participants were selected using a simple random sampling technique.

### Data collection tools and procedures

A pretested structured questionnaire was used. The questionnaire adopted from previous literature [12, 20]. The questionnaire had three parts namely socio-demographic characteristics, obstetric /gynecological related characteristics and presence of primary dysmenorrhea, intensity and their impacts. Pain associated with menstruation without any pelvic pathology was considered as primary dysmenorrhea.

The irregular monthly menstrual cycle is taken as longer/heavy bleeding than a usual menstrual cycle [21]. Using a verbal report from the Numeric Rating Scale (NRS); the intensity of primary dysmenorrhea related pain was considered as no pain (NRS = 0), mild pain (NRS = 1–3), moderate pain (NRS = 4–6), and severe pain (NRS = 7–10) [22]. The data were collected by five nurses (three data collectors and two supervisors) from May 1–10/2017 using a self-administered technique.

### Data processing and analysis

Data were checked, coded, and entered into Epi Info version 7 and exported to SPSS version 20 for analysis. Descriptive statistics such as frequency and percentage were used. Tables and bar graphs were used to display the findings. A binary logistic regression model was used to identify factors associated with primary dysmenorrhea. Variables whose *p*-value≤0.2 in the bivariable logistic regression analysis were taken into multivariable logistic regression analysis. For the bivariable logistic regression; Crude Odds Ratio (COR) and 95% CI, and for the multivariable logistic regression; Adjusted Odds Ratio (AOR) and 95% CI were calculated. Variables having a *p*-value < 0.05 in the multivariate logistic regression model were considered as statistically significant. The backward stepwise logistic regression analysis method was used. Hosmers and Lemishow goodness of fit test were done.

## Results

### Socio-demographic characteristics of respondents

A total of 459 female students were enrolled in the study with a response rate of 96.29%. Among study participants, 232(52.5%) were aged less than 18 years, 286(64.7%) were grade eleven, 398(90%) were single, and 403(91.2%) were Orthodox Christian followers. More than threefold (94.3%) of study participants were Amhara by ethnicity. The majority (86.7%) of the respondents were urban dwellers and 370(83.7%) were lived with their family home. Regarding family education, more than half of respondent mothers’ were illiterate and 294(66.5%) were housewives by their occupational status. Above one–third (40%) study participants carry out sports activity (Table 1).

**Table 1:** Socio-demographic characteristics of female students at Gondar town preparatory school Northwest Ethiopia, 2017, (*n* = 442)

Variables	Category	Frequency (n)	Percent (%)
Age in year	≥18	210	47.5
	< 18	232	52.5
Student grade	11^th^	286	64.7
	12^th^	156	35.3
Religion	Orthodox Christian	403	91.2
	Muslim	28	6.3
	Protestant	11	2.5
Marital status	Single	398	90
	Married	39	8.8
	Divorced	4	0.9
	Widowed	1	0.2
Ethnicity	Amhara	417	94.3
	Qimant	13	3
	Tigray	12	2.7
Origins of residence	Urban	383	86.7
	Rural	59	13.3
Place of residence	At family home	370	83.7
	With other relatives	37	8.4
	At dormitory	35	7.9
Mother’s education	Illiterate	228	51.6
	Primary school	61	13.8
	Secondary school	95	21.5
	College and above	58	13.1
Mother’s Occupation	Government employed	88	19.9
	Housewife	294	66.5
	Merchant	50	13.6
Sport activity	Yes	177	40
	No	265	60

### Obstetrics and gynecology related characteristics

Among study participants, 250(56.6%) experienced 1st menstruation at the age group of 12–14 years. Nearly three-fourths (73.5%) of study participants had regular monthly menstrual cycle pattern and the vast majority (91.9%) of study participants reported menstrual bleeding duration ≤7 days. One hundred eighty- three (41.4%) of study participants reported that they had a positive family history of dysmenorrhea (Table 2).

**Table 2:** Obstetric and gynecological related characteristics of female students at Gondar town preparatory school Northwest Ethiopia, 2017, (*n* = 442)

Variables	Category	Frequency (n)	Percent (%)
Age at menarche	12–14	250	56.6
	≥15	192	43.4
Monthly menstrual cycle	Regular	325	73.5
	Irregular	117	26.5
Menstrual bleeding duration (in days)	≤7	406	91.9
	> 7	36	36.1
Estimated amount of menstrual flow /by no. of pads	≤2	288	65.2
	3–4	141	31.9
	> 4	13	2.9
Any family planning method?	Yes	30	6.8
	No	412	93.2
Type of family planning used	Depo provera	10	33.3
	Pills	8	26.7
	Implanol	7	23.3
	IUCD	5	16.7
Family history of dysmenorhea	Yes	183	41.4
	No	259	58.6
Circumcision history	Yes	38	8.6
	No	404	91.4
Abortion history	Yes	10	2.3
	No	432	97.7

*IUCD*; Intrauterine Contraceptive Device

### Prevalence, intensity, and impact of primary dysmenorrhea

**Fig. 1:**
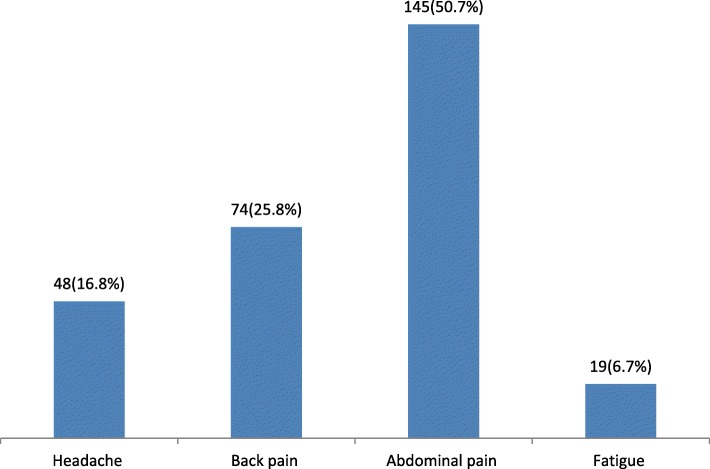
Common complaining symptoms associated with primary dysmenorrhea among female students at Gondar town preparatory school, Northwest Ethiopia, 2017

The prevalence of primary dysmenorrhea was found to be 64.7%(95% CI; 60.2, 69.2%) of which nearly half (50.7%) of them complain about lower abdominal pain (Fig. 1). The intensity of pain during menstruation was 83(29%), 174(60.8%), and 29(10.2%) reported having mild, moderate, and severe menstrual pain respectively. Seventy-three (25.5%) used analgesic medications. Paracetamol 44(60.3%), diclofenac 15(20.5%), and Ibuprofen 14(19.2%) were used to relieve the pain. Absenteeism from school 186(65%), limited activity daily living 57(20%) and anxiety 43(15%) were the impact of menstrual pain reported by study participants.

### Factors associated with primary dysmenorrhea

All variables with *p*-value ≤0.2 in the bivariable logistic regression analysis were taken into multivariable analysis. Having an irregular monthly menstrual cycle and a positive family history of dysmenorrhea were significantly associated with primary dysmenorrhea in multivariable logistic regression analysis (Table 3).

**Table 3:** Factors associated with primary dysmenorrhea among female students at Gondar town preparatory school Northwest Ethiopia, 2017, (*n* = 442)

Variables	Primary dysmenorrhea		COR, 95% CI	AOR, 95% CI
	Yes	No		
Place of residence				
At family home	239	131	1.216 (0.599,2.471)	1.442 (0.650,2.471)
With other relatives	26	11	1.576 (0.593,4.185)	2.105 (0.725,6.106)
At dormitory	21	14	1	1
Age at menarche				
12–14	164	86	1.094 (0.739,1.621)	1.046 (0.672,1.629)
≥ 15	122	70	1	1
Monthly menstrual cycle				
Regular	200	125	1	1
Irregular	86	31	1.734 (1.086,2.767)	1.701 (1.019, 2.839)^a^
Menstrual bleeding duration				
≤ 7 days	262	144	1	1
> 7 days	24	12	1.099 (0.534,2.263)	1.081 (0.467,2.502)
Family history of menstrual pain				
Yes	153	30	4.832 (3.047,7.661)	5.188 (3.214,8.373)^a^
No	133	126	1	1
Circumcision history				
Yes	28	10	1.584 (0.748,3.354)	1.531 (0.681,3.443)
No	258	146	1	1

^a^statistically significant at *p*-value < 0.001

## Discussion

The prevalence of primary dysmenorrhea was found to be 64.7% (95% CI; 60.2, 69.2%). Lower abdominal pain (50.7%), back pain (25.5%), headache (16.8%), and fatigue (6.7%) were the commonly reported symptoms among study participants. Nearly 61% reported moderate intensity of menstrual pain. Sixty-five percent of study participants reported that absenteeism from school is the main impact of menstrual pain. The finding of this study is in line with a study conducted at Debre Tabor, Ethiopia 62.3% [23] and Chandigarh, India 61.33% [24]. The finding of the current study was higher than a study in Canada (60%), Japan (46,5%) and (54.4%)**,** Tbilisi, Georgia (52.07%), and Southern India (45%) [25–29]. The variation is due to the assessment tool, method of data collection such as interviewing using a cell phone, use of clinical assessment like ultrasound and laboratory test, and sample size used.

To the contrary, the finding of the current study was lower than a study conducted in Iran (89.1%), Turkey (72.7%), Debre Birhan University (85.4%) and University of Gondar Ethiopia (77.6%), Mansoura, Egypt (75%), Pakistan (78%), Iran (73.2%), and Saveetha University (70.4%) [26, 30–35]. This is due to the study setting, age of study participants, and most of the studies did not differentiate whether the dysmenorrhea is primary or secondary.

In this particular study, a positive family history of dysmenorrhea and irregular menstrual cycle are the determinant factors of primary dysmenorrhea. The study revealed that females who had a positive family history of dysmenorrhea were five times more likely to develop primary dysmenorrhea [AOR = 5.19, 95% CI: 3.21, 8.37] compared to those who had no family history of dysmenorrhea. This is supported by a study in Turkey [31], Iran [30], Eastern, Benin [36], Serbia [37], and Australia [38]. This is related to genetic linkage between the mother and the child that makes to develop menstrual pain. Studies suggest that genetic background influences the severity of dysmenorrhea [39].

Having an irregular monthly menstrual cycle is the determinant factor of primary dysmenorrhea. Female students who had irregular monthly menstrual cycle is nearly two times more likely to develop primary dysmenorrhea compared to those having regular monthly menstrual cycle [AOR = 1.701, 95% CI: 1.02,2.84]. No studies showed the relationship between the irregular monthly menstrual cycle and menstrual pain but it is associated with prostaglandin secretion. The author only used reports obtained from the respondents, no clinical examination was done. Since most studies conducted dysmenorrhea as a combined manner (primary and secondary dysmenorrhea) the author forced to discuss the current study with those studies. Further research is needed to find out the real cause of primary dysmenorrhea.

## Conclusions

The prevalence of primary dysmenorrhea among female students found to be high. Irregular monthly menstrual cycle and positive family history of dysmenorrhea are determinant factors of primary dysmenorrhea. Absenteeism from school was the main impact of primary dysmenorrhea. Hence, researching primary dysmenorrhea significantly improves its impact by educating females about the management of primary dysmenorrhea.

## Data Availability

The raw data would not be provided for the reason of protecting patients’ confidentiality but, the summary data are available in the main document.

## Abbreviations

AOR: Adjusted Odds Ratio
CI: Confidence Interval
COR: Crude Odds Ratio
IUCD: Intrauterine Contraceptive Device
NRS: Numeric Rating Scale
